# *Periplaneta americana* Extracts Promote Skin Wound Healing via Nuclear Factor Kappa B Canonical Pathway and Extracellular Signal-Regulated Kinase Signaling

**DOI:** 10.1155/2017/5821706

**Published:** 2017-05-23

**Authors:** Qin Song, Qiheng Gou, Yuxin Xie, Zhen Zhang, Chaomei Fu

**Affiliations:** ^1^College of Pharmacy and Bioengineering, Chengdu University, Chengdu, Sichuan 610106, China; ^2^State Key Laboratory of Biotherapy/Collaborative Innovation Center of Biotherapy, West China Hospital, Sichuan University, Chengdu 610041, China; ^3^Departments of Head and Neck and Mammary Gland Oncology and Medical Oncology, Cancer Center and State Key Laboratory of Biotherapy, Laboratory of Molecular Diagnosis of Cancer, West China Hospital, Sichuan University, Chengdu, Sichuan 610041, China; ^4^College of Pharmacy, Chengdu University of Traditional Chinese Medicine, Chengdu, Sichuan 610106, China

## Abstract

*Periplaneta americana *extracts (PAEs) exhibit wound healing properties. However, the underlying molecular mechanisms are not well understood. Here, we treated human skin fibroblasts (HSF) with PAE and the proliferation was determined by 3-(4,5-dimethylthiazol-2-yl)-2,5-diphenyltetrazolium bromide (MTT) assay. The wound healing and transwell migration assays were used to detect cell migration. Nuclear factor kappa B (NF-*κ*B) and extracellular signal-regulated kinase (ERK) pathways were analyzed by Western blot (WB). Immunofluorescence staining was used to detect the key molecular localization in the cells. The results showed that PAE enhanced the proliferation and migration of HSF cells. The expression and activation of key proteins such as RelA and p-ERK were increased in NF-*κ*B and ERK pathways followed by nuclear translocation. In vivo, both WB and immunohistochemical (IHC) staining showed that PAE enhanced p-I*κ*B*α* and p-ERK activation and the nuclear translocation of RelA. Our study suggests that the protective function of PAE is mediated via enhanced NF-*κ*B and ERK signaling.

## 1. Introduction

Skin wound healing is a complex process including cell proliferation, migration, and matrix synthesis. It is commonly divided into three stages including: (1) inflammatory cell phase, (2) cell proliferation, and (3) tissue remodeling [1]. Various types of cells including epithelial and mesenchymal cells and fibroblasts play a key role in wound healing [2]. Thermal injury of skin is caused by tissue lesions following exposure to flames, hot surfaces and liquids, extreme cold, chemicals, radiation, or friction [3]. Even with improved prognosis and therapeutic intervention via biological skin substitutes, burns represent an important cause of mortality.

*Periplaneta americana* exhibits therapeutic effects in wound healing. In 1985, the* Periplaneta americana *extract (PAE), also designated as W11-a12 or Kangfuxin, was first used clinically. Subsequently, it has been widely used in China to heal severe ulcers and burns. It is administered intravenously and orally or directly applied on the wounds topically [4, 5]. The mechanisms underlying the healing effect of PAE are not fully established. Early studies suggested that PAE promoted healing in rats sustaining combined radiation (6 Gy) [3] and wound injury [6]. In the present study, we focused on the molecular mechanisms of PAE applied topically on skin wounds in mice following to thermal injury.

ERK belongs to the mitogen-activated protein kinase (MAPK) family. ERK1 and ERK2 form a central component in the MAPK cascade and play a crucial role in signal transduction from surface receptors to the nucleus. Activated ERK dimers translocate to the nucleus and regulate several gene transcriptions, such as Elk-1, ATF, NF-*κ*B, Ap-1, and c-fos, leading to cell proliferation and differentiation, cytoskeletal structure, cell apoptosis, and other biological reaction [7, 8]. The biological effect, in turn, dictates whether ERK-expressing cells enter a program of cell death, survival, or differentiation [9, 10].

NF-*κ*B is a transcription factor regulating the expression of multiple genes and cellular functions, including migration and survival [11]. NF-*κ*B activation is regulated by negative feedback mediated by I*κ*B, an inhibitor that binds to NF-*κ*B, but undergoes ubiquitin-mediated proteasomal degradation, releasing NF-*κ*B for nuclear translocation and transcription [12]. NF-*κ*B triggers cell proliferation and migration. Abnormal NF- *κ*B expression induces autoimmune disease, chronic inflammation, metabolic disease, and cancer [13]. Early evidence reveals three separate pathways: the canonical NF- *κ*B pathway; the alternative NF- *κ*B pathway; and an independent pathway [14, 15]. The canonical pathway is involved in the fibroblast migration and progression of wound healing. Furthermore, NF-*κ*B is a redox-sensitive transcription factor acting as a sensor of oxidative stress [16]. Thermal injury induces local tissue hypoxia. We hypothesize that PAE treatment improves NF-*κ*B signaling via activation of wound healing. Thus, in the present study, we investigated the biological function and mechanisms of PAE in human skin fibroblast and rat skin injury models to facilitate the clinical application of PAE.

## 2. Materials and Methods

### 2.1. Cell Lines

Human skin fibroblast (HSF) cell line purchased from the American Type Cell culture/ATCC CRL-2522™ was cultivated in DMEM with 10% fetal bovine serum (Gibco Life Science, Grand Island) and 1% penicillin-streptomycin (Sigma, V900929) at 37°C in a humidified incubator with 5% carbon dioxide.

### 2.2. Preparation of PAE

**Figure 1:**
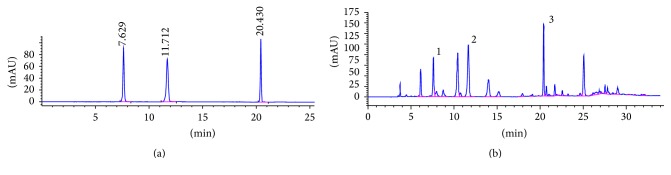
Chromatographic separation of PAE. (a) HPLC chromatogram of authentic standards tested. (b) HPLC chromatogram of PAE at 254 nm: peak 1, uracil (1.140 mg/g); peak 2, hypoxanthine (4.257 mg/g); and peak 3, inosine (8.158 mg/g). Identification was based on retention time and UV spectra compared with commercial standards.

*P. americana* was obtained from the Good Agriculture Practice (GAP) breeding base, Sichuan, China. The powdered dried* P. americana* (200 g) was extracted with 90% EtOH (1.2 L) twice at 80°C. After solvent evaporation, the ethanol extract was recovered. The extract (20 g) was suspended in water (200 mL) at 80°C. After filtration through 0.22 *µ*m filter membranes at appropriate concentrations, it was stored at −20°C until use. The HPLC-diode array detector (HPLC-DAD) was used to study* P. americana* extraction. The compounds in PAE were analyzed (Figure 1) using Diamonsil C18 (250 × 4.6 mm; 5 *µ*m) as the chromatography column. The optimized mobile phase consisted of solvent A (3% v/v methanol in water containing 0.07% v/v acetic acid) and solvent B (methanol). The following gradient of time (min)/mobile phase A (%)/mobile phase B (%) was used: 0.0/100/0, 10/100/0, 20/70/30, 21/50/50, and 35/0/100, at a flow rate of 0.6 mL/min at 25°C and detection wavelength 254 nm with 10 *μ*L injection volume.

### 2.3. MTT Cell Proliferation Assays

The in vitro cell proliferation induced by PAE was determined using MTT assay. Briefly, cells were seeded in a volume of 200 *µ*L (3,000 cells/well) on 96-well plates after cultivation with different concentrations of PAE. The culture medium containing serum was replaced by MTT every 24 h. A final MTT concentration of 0.5 mg/mL was added to the wells followed by incubation for 4 h at 37°C. The supernatant was discarded and replaced with DMSO (150 *µ*L/well). The optical densities (OD) were measured at 570 nm with a NOVOstar microplate reader. The experiment was repeated in triplicate. The viable concentration was calculated using GraphPad Prism 5.0.

### 2.4. Transwell Migration Assay

To assay the migratory behavior of HSF cells following PAE treatment in the Transwell Milicells (8 *μ*m pore size, Millipore, USA), a 90% confluent T-25 flask of HSF cells was treated with or without PAE (0.3125 mg/mL). A 600 *µ*l 10% FBS growth medium was added to the lower chamber, followed by trypsinization using standard procedures. The final pellet was resuspended in 2 mL of serum-free medium (SF-EMEM) and seed cells (2 × 10^4^/cells) in the upper chamber. After incubation for 24 h, the chambers were fixed with 4% paraformaldehyde for 30 min and stained with hematoxylin for 15 min. We counted the cells using optical microscope.

### 2.5. Cell Scratch Tests

Cell scratch test is particularly appropriate for studies investigating the effect of cell-matrix and cell-cell interactions on cell migration. HSF were seeded in the 6-well plate and the 10% FBS growth medium containing the serum-free medium supplemented with the PAE (0.3125 mg/mL) was grown to 90% confluence. After treatment for 48 h, the culture medium was removed and the monolayers were scratched using a 200 *μ*L pipette to create a uniform cell-free wound area. Debris was removed by gently washing with sterile PBS. Cell movement into the wound area was monitored and photographed at 0, 24, and 48 h using an optical microscope.

### 2.6. Western Blot

Total protein extracts (30 to 50 *μ*g) from cells lysates were prepared. Each sample was subjected to electrophoresis on 12% SDS-polyacrylamide gels. Then, the protein was blotted onto a PVDF membrane (Millipore, Billerica, MA) at 230 mA for 2 h. Primary antibodies against IKK *β* (1 : 1,000; Cell Signaling Technology, Beverly, MA, USA), p-I*κ*B*α* (1 : 1,000; Cell Signaling Technology, Danvers, MA, USA), I*κ*B*α* (1 : 1,000; Cell Signaling Technology, Beverly, MA, USA), RelA (1 : 1,000; Cell Signaling Technology, Beverly, MA, USA), p-ERK (1 : 1000; Cell Signaling Technology, Beverly, MA, USA), ERK (1 : 1000; Cell Signaling Technology, Beverly, MA, USA), and *β*-actin (1 : 1,000; Sigma-Aldrich, St. Louis, MO, USA) were used, according to the manufacturer's instructions. After washing the membrane, the secondary antibody (HRP-conjugated anti-mouse/rabbit IgG) was used for detection. The bands were visualized with the ECL detection system.

### 2.7. Immunofluorescence Staining

After seeding the cells on sterile slides for 24 h, different doses of PAE were added for 48 h. Each group of HSF cells was washed twice with PBS and fixed with 4% paraformaldehyde (pH 7.4) in 6-well plates and incubated with 0.5% Triton X-100 for 30 min at room temperature, followed by blocking with 5% BSA for 1 h. The slides were incubated with the following primary antibodies: RelA (dilution 1 : 100; Cell Signaling Technology, Beverly, MA, USA), phospho-ERK (Cell Signaling Technology, Beverly, MA, USA), and ERK (Cell Signaling Technology, Beverly, MA, USA) overnight at 4°C. The cells were incubated with the corresponding fluorescent dye-conjugated secondary antibodies (dilution 1 : 200; Cell Signaling Technology, Beverly, MA, USA) at 37°C for 1 h and protected from light. The cells were visualized using fluorescence microscopy.

### 2.8. Mouse Model of Thermal Burn

Eight healthy adult C57 male mice were purchased from the West China School of Preclinical and Forensic Medicine, Sichuan University, China. All the experiments were conducted according to the Guide for the Care and Use of Laboratory Animals at the Animal Experimental Center of Sichuan. Initially, 8 animals were weighed and intramuscularly injected with atropine sulfate (0.04 mg/kg). After 10 minutes, they were injected with anesthetic combination of 10% ketamine (90 mg/kg) and 2% xylazine (10 mg/kg) intramuscularly. When the animals properly anesthetized, their backs were treated with 1% polyvinylpyrrolidone iodine. Thermal injuries were created with a solid aluminum bar (*φ*10 mm) previously heated in boiling water (100°C). The bar was maintained symmetrically in contact with the skin on the dorsal flank for 15 s. The pressure exerted on the animal skin corresponded to the mass of 50 g. Immediately after the procedure, analgesia with sodium dipyrone (40 mg/kg) was performed intramuscularly and was maintained with sodium dipyrone (200 mg/kg) in the drinking water for three consecutive days. The left dorsal skin was wiped with PAE (5 mg/mL), while the right was treated with equal amounts of normal saline. In the course of 21-day treatments, wound healing rates were measured at day points 0, 7, 14, and 21 after treatment. The wound healing rates were measured and the complete wound healing time was calculated using the formula: healing rate = original wound area/original wound area [17]. Mice were sacrificed after 21 days.

### 2.9. Immunohistochemical and Immunocytochemical Staining

Briefly, the skin tissues were fixed in formalin and embedded in paraffin. Consecutive paraffin sections (4 *μ*m-thick) of tissue samples were prepared and incubated overnight at 4°C with primary antibodies, followed by incubation with peroxidase-labeled polymer conjugated to goat anti-rabbit immunoglobulins (EnVision/HRP, Dako, Denmark). All the IHC assays were carried out according to the manufacturer's instructions.

### 2.10. Statistical Analyses

Statistical analyses were performed using SPSS 11.5 (SPSS Inc., Chicago, IL, USA) or Prism 6.0 (GraphPad Software, La Jolla, CA, USA). Quantitative data were evaluated with a two-tailed Student's *t*-test, and one-way analysis of variance (ANOVA). Differences were considered statistically significant at *p* < 0.05.

## 3. Results

### 3.1. PAE Promotes Cell Proliferation In Vitro

**Figure 2:**
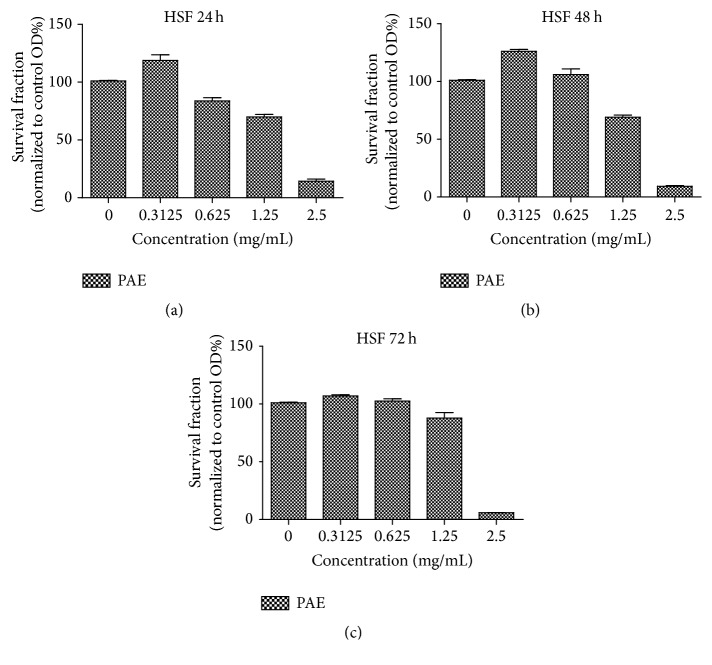
MTT assay using different doses of PAE on HSF at different time points. (a) The survival fraction of different doses of extract on HSF at 24 h (normalized to control group); (b) the survival fraction of different doses of extract on HSF at 48 h (normalized to control group); (c) the survival fraction of different doses of extract on HSF at 72 h (normalized to control group).

MTT cell proliferation assay was used to detect the appropriate concentration of PAE and the time point on the HSF cell line at early passages (passage 8–10). The assays performed at 24 h, 48 h, and 72 h showed that the low (0.3125 mg/mL) dose of the PAE promoted cell growth (Figure 2), especially at 48 h of treatment (*p* < 0.05; one-way ANOVA). Interestingly, the data presented in Figure 2 indicated that the PAE at higher concentration (1.25 mg/mL) could inhibit the cell proliferation. Above all, we selected the optimal concentration of 0.3125 mg/mL with the time of 48 h treatment in subsequent assay.

### 3.2. PAE Facilitates Migration of HSF

Cell migration into a “wound” created on a monolayer of cells revealed the effects of wound healing. Denudation of part of the HSF induced epidermal cell migration to close the wound with loosely connected cell populations, which also mimicked the behavior of cells during migration in vivo [18].

**Figure 3:**
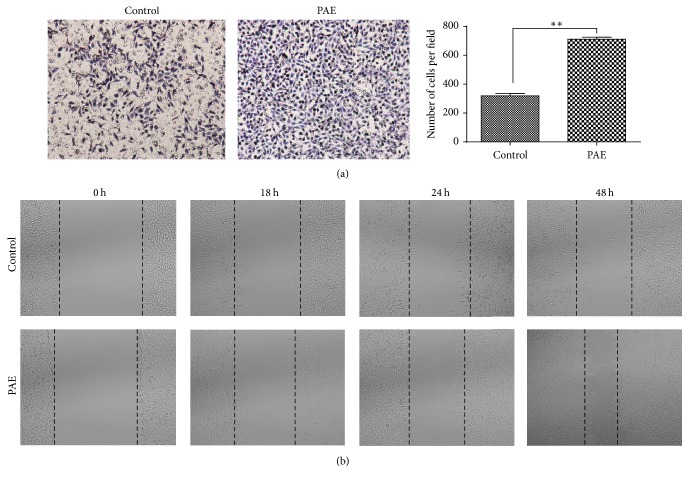
Effect of PAE on cell migration in HSF cells. The differentially cultured HSF (0.3125 mg/mL) for 48 h. (a) Transwell migration assay: images obtained at 24 h after HSF were incubated in Milicells 24 h (left); the migratory cells per visual field (100x) were counted and expressed as the average numbers. Data represent mean ± SD. ^*∗∗*^*p* < 0.01 versus control group (right). (b) Cell scratch test: images obtained at 0, 18, 24, and 48 h after scratch formation.

To determine whether PAE promoted HSF cell migration in skin, we performed transwell and wound healing assays. Human skin fibroblasts were treated with PAE at the final concentration of 0.3125 mg/mL for 48 h. As shown in Figure 3(a), cells treated with PAE showed 2-fold migration efficiency compared with control (*p* < 0.01). Similar to the transwell assay, the cell scratch test also significantly improved the migration of wound closure in the PAE-treated group of HSF cells (Figure 3(b), *p* < 0.01).

### 3.3. PAE Enhanced NF-*κ*B Canonical Pathway and ERK Phosphorylation

**Figure 4:**
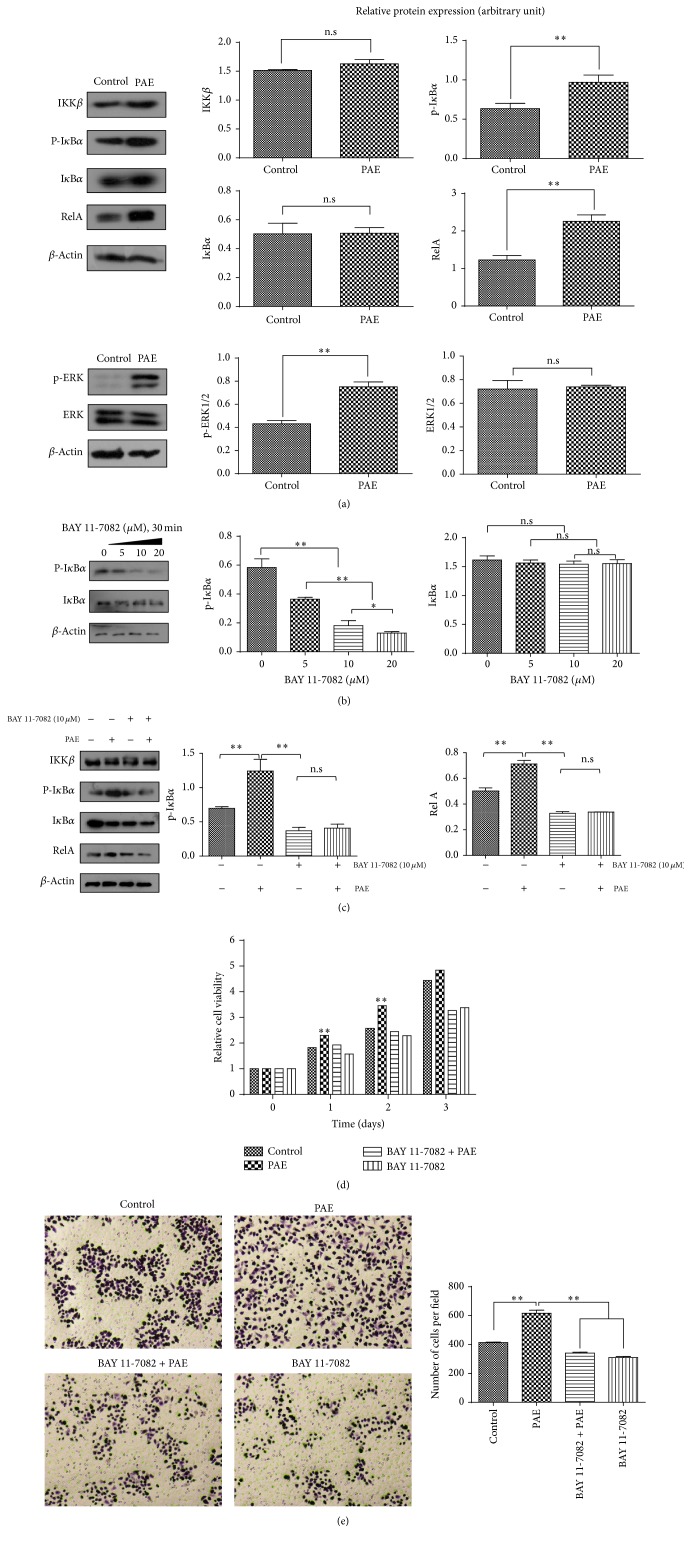
PAE affected NF-*κ*B canonical and ERK pathways. HSF cells were differentially cultured for 48 h, and the protein expression of (a) NF-*κ*B canonical pathway and ERK pathway varied; (b) NF-*κ*B canonical pathway proteins in the presence of BAY 11-7082; (c) NF-*κ*B pathway expression in the presence of BAY 11-7082 and PAE. Actin was used as a loading control. Cells were incubated with a medium containing PAE at the indicated concentrations for 48 h after pretreatment with BAY 11-7082 (10 *μ*M) for 30 min. Cell proliferation (d) was determined by MTT and (e) migration was tested by transwell assay. Data represent mean ± SD. ^*∗*^*p* < 0.05; ^*∗∗*^*p* < 0.01 versus control group.

Compared with the control, the expression of downstream RelA in the canonical NF- *κ*B pathway was significantly higher in the PAE-treated cells. Interestingly, we found that the level of ERK phosphorylation was significantly improved while the level of ERK total protein scarcely changed (*p* < 0.05) (Figure 4(a)). These suggested that the PAE enhanced NF-*κ*B canonical pathway and ERK pathway. Furthermore, we determined the molecular mechanism of PAE in the activation of NF-*κ*B pathway using BAY 11-7082, a complete and specific NF-*κ*B pathway inhibitor (Figure 4(b)) [13]. The results suggested that the expression of p-I*κ*B*α* was gradually decreased depending on the dose of BAY 11-7082. Further, the activities of NF-*κ*B pathway were suppressed. We added the PAE (0.3125 mg/mL final concentration) after the inhibition of NF-*κ*B pathway using the BAY 11-7082. After treatment with PAE for 48 h, we found that the reintroduction of PAE failed to restore the expression of p-I*κ*B*α* suggesting that NF-*κ*B pathway was still suppressed (Figure 4(c)). Moreover, as shown in Figures 4(d) and 4(e), the PAE-induced cell growth and migration were prevented by pretreatment with BAY 11-7082 in HSF cells. The result confirmed that the PAE analogues promoted HSF migration and proliferation by activating the NF-*κ*B pathway.

### 3.4. PAE Increases RelA and Phosphorylation of ERK Nuclear Translocation

**Figure 5:**
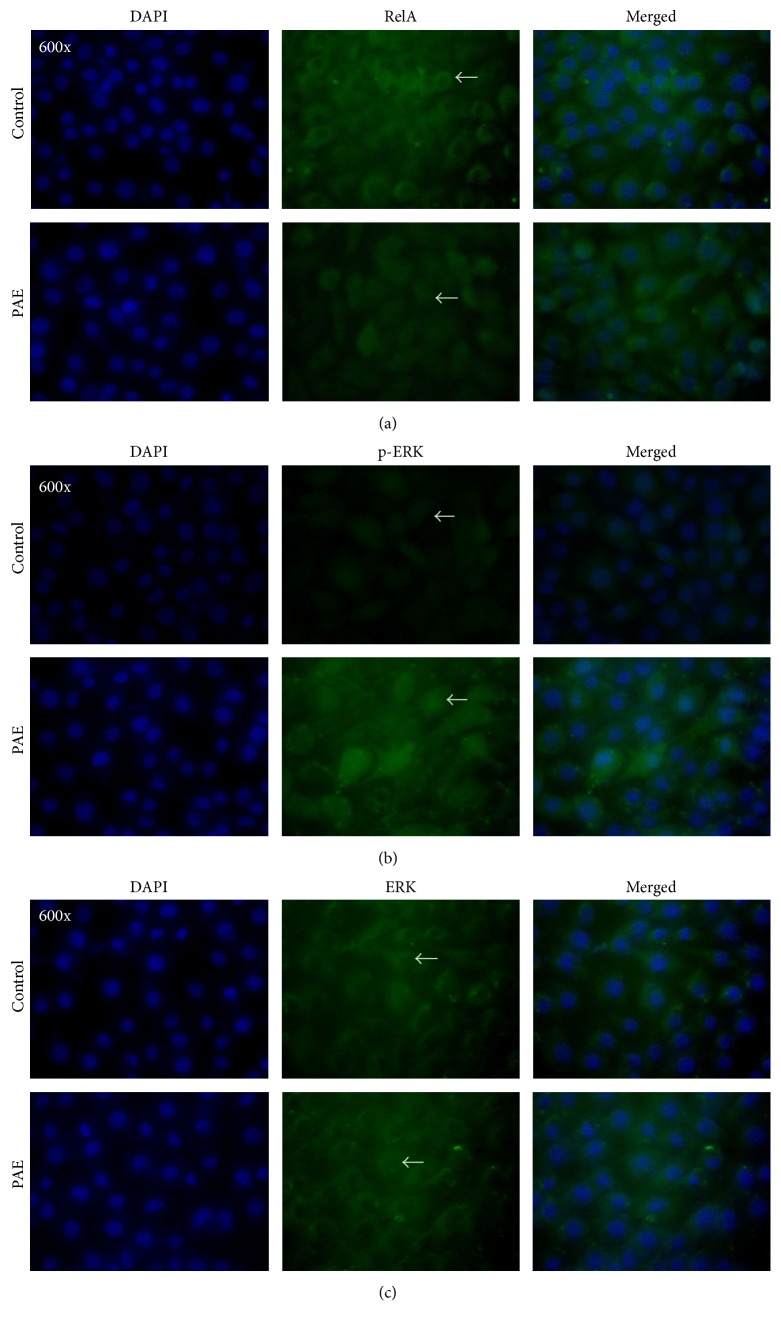
Immunofluorescence analysis of the spatial localization of the NF-*κ*B canonical and ERK pathways. Cells were treated with PAE for 48 h, fixed, and incubated to determine the immunofluorescence. The antibodies against (a) RelA, (b) p-ERK, and (c) ERK presented green fluorescence, whereas a blue fluorescent signal was generated by DAPI staining of the cell nuclei. Areas of overlap between the green and the blue fluorescence appeared as merged images.

As illustrated in Figure 5(a), the antibody against RelA showed green fluorescence whereas the nucleus was stained blue by DAPI. Areas of overlap in the merged images presented aquamarine color. The control group exhibited green fluorescence in the cytoplasm. During the PAE treatment, the green fluorescence was evenly distributed, and the aquamarine area in the merged image was increased suggesting translocation of RelA from the cytoplasm to the nucleus. Similar results were detected in the p-ERK protein (Figure 5(b)). However, ERK translocation occurred barely (Figure 5(c)). PAE increased the protein levels along with the nuclear translocation of RelA and p-ERK proteins.

### 3.5. PAE Promoted Wound Healing of Cutaneous Thermal Burn In Vivo

Based on the effect of PAE on migration and proliferation in vitro, we hypothesized that the PAE improves skin wound healing in vivo.

Briefly, we created a deep second-degree thermal burn in C57 mice, as described in Materials and Methods. Originally, we failed to distinguish the skin lesions until swelling and ulceration were detected during the following days. The burn was wiped with PAE. The treated wound showed significant healing.

**Figure 6:**
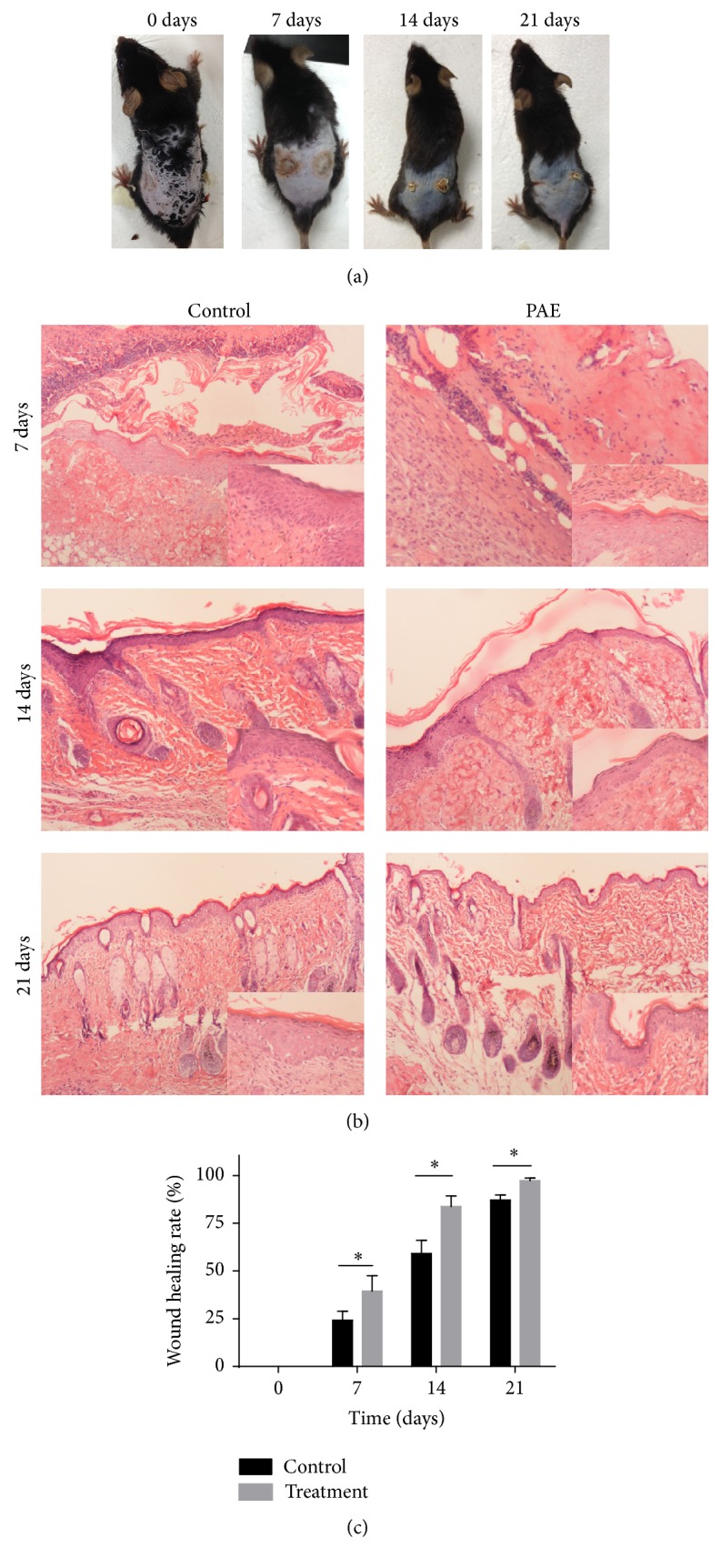
PAE affected wound healing in cutaneous thermal burns in vivo. In the C57 mouse model of thermal injury, the left wound of the dorsal flank was treated with PAE (5 mg/mL) while the right side treated with normal saline served as the daily control. (a) Representative images of thermal injury on days 0, 7, 14, and 21; (b) histological HE staining of differentially treated tissues on days 0, 7, 14, and 21; (c) healing rate of thermal wound. ^*∗*^*p* < 0.05 versus control group.

Microscopic, immunohistochemical, and immunocytochemical staining indicated epithelial repair, follicular regeneration, and fibrous tissue formation following PAE treatment. The healing rate and healing phase showed significant differences between the PAE-treated wound and control wound. The healing rate of PAE-treated wound was higher than that of the control during the healing phase (Table 1 and Figure 6, *p* < 0.05). In general, the results demonstrated that PAE greatly accelerated the healing of burn wounds.

### 3.6. PAE Enhanced NF-*κ*B Pathway and ERK Phosphorylation In Vivo

**Figure 7:**
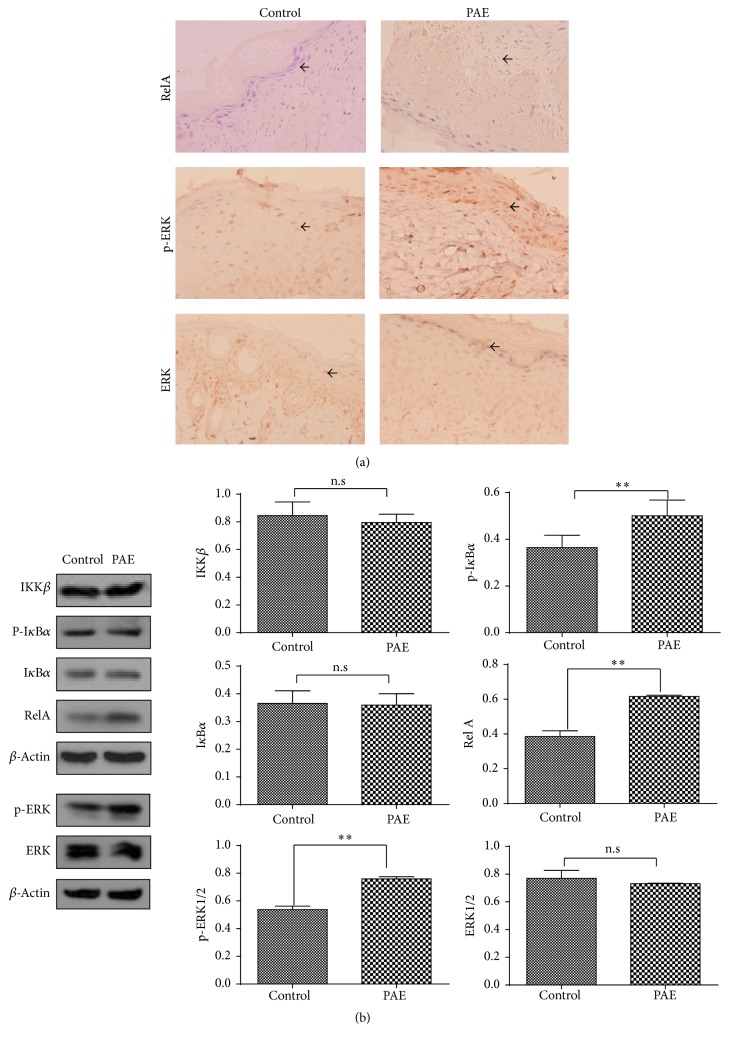
NF-*κ*B canonical pathway and ERK pathway activities in vivo. (a) Molecular expression of NF-*κ*B canonical pathway and ERK pathway in PAE-treated and control tissues was assayed by IHC, and the representative images are shown (400x). (b) Western blot of protein expression in NF-*κ*B canonical and ERK pathways. Actin was used as a loading control. Data represent mean ± SD. ^*∗∗*^*p* < 0.01 versus control group.

Tissue microarrays were constructed using the samples collected regularly from the wound margins with different treatments using the method described. Immunohistochemical staining (IHC) further confirmed the regulatory mechanism of NF-*κ*B and ERK pathways underlying the PAE effect during the healing phase. Specifically, the level of RelA in NF-*κ*B pathway was significantly improved in the treated group, and the p-ERK staining was increased while that of the ERK total protein was barely altered (Figure 7(a)). Simultaneously, the Western blot suggested the role of RelA in PAE-treated wound and the expression of upstream IKK *β* and I*κ*B*α* showed no significant changes between the different groups. The expression of ERK phosphorylation was enhanced while that of total ERK showed no changes (Figure 7(b)). Thus, the PAE promoted wound healing by enhancing NF-*κ*B and ERK pathway activities in vivo.

## 4. Discussion

The therapeutic role of PAE in wound healing has been demonstrated clinically [19–21]. However, due to the complex chemical composition, its potential molecular mechanisms are still unclear. In the current study, we extracted and purified the products from* P. americana* and analyzed the extract with greater efficacy for further study. We detected the effect of PAE on the proliferation and migration of human skin fibroblasts in vitro and in healing of thermal injury using the mouse model [4, 19]. Results indicate that PAE promoted wound healing via NF-*Κ*B signaling in vitro and in vivo.

Wound healing is a well-orchestrated biological event composed of three distinct but overlapping phases: inflammation, proliferation, and remodeling. Fibroblast is one of the major skin components and therefore regarded as the important determinants of wound healing efficiencies [22]. In our study, we found that PAE promoted proliferation and migration of HSF [23]. To establish the pharmacological effects of PAE and validate the optimal extraction process, the HSF were used in the in vitro wound healing model for evaluation of the cellular and molecular effects of PAE. The fibroblasts enhanced proliferation and migration after treatment with PAE (0.3125 mg/mL) for 48 h. In addition, PAE significantly increased the healing rates and reduced the healing time by enhancing epithelial repair, follicular regeneration, and fibrous tissue proliferation following cutaneous thermal injury in vivo [24–26].

The expression of growth mediators is modulated by NF-*κ*B pathway. NF-*κ*B plays a critical role in regulating the immune response to extracellular stimuli. It is normally sequestered in the cytoplasm by a family of inhibitory proteins known as inhibitors of NF-*κ*B (I*κ*Bs) [16, 27]. Activation of NF-*κ*B, releases RelA from its inhibitory protein I*κ*B-*α*, followed by nuclear translocation to trigger the transcription of specific target genes such as TNF-*α*, IL-1*β*, and IL-6 [23, 28]. To determine the molecular mechanism of NF-*κ*B activation, we tested the expression of upstream IKK *β*, P-I*κ*B*α*, and I*κ*B*α* and downstream RelA activation. The results showed that PAE stimulation dramatically increased the phosphorylation of I*κ*B*α* and RelA proteins. However, PAE-induced I*κ*B*α* and RelA activation was significantly blocked by pretreatment with BAY 11-7082. Meanwhile, we found similar results in the cutaneous thermal injury in vivo. Collectively, our results suggested that PAE promoted the migration and proliferation in vitro and wound healing in vivo via NF-*κ*B activation.

Simultaneously, we hypothesized that PAE might activate more than one pathway resulting in HSF proliferation and migration. We found an increase in the phosphorylation and nuclear translocation of p-ERK despite stable levels of total ERK. Phosphorylation and nuclear translocation of ERK1 and ERK2 are critical for gene transcription and expression. Therefore, the kinetics and localization of ERK1/2 are intrinsically linked. ERK1 and ERK2 are the most thoroughly studied in the ERK family and involved in a wide range of physiological processes, including the regulation of cell meiosis, mitosis, and anaphase. A variety of stimuli such as growth factors and cytokines are associated with ERK1/2 pathway activation. ERK activation determines its cellular proliferation and migration [29]. In this study, PAE acts as a stimulus to phosphorylate ERK1/2, resulting in skin wound healing.

## 5. Conclusion

In summary, PAE promotes wound healing in vitro and in vivo in experimental models. These processes are closely correlated with the increased activation of NF-*κ*B and ERK signaling pathways. Furthermore, the enhanced activities of NF-*κ*B and ERK pathways may represent major molecular targets of PAE, which accelerate wound healing by upregulating the expression of a series of genes involved in cell proliferation, fibrogenesis, reepithelialization, and remodeling [2, 29, 30].

## Figures and Tables

**Table 1:** Healing rates at different time points after burn (%).

Group	7 d	14 d	21 d
Control	24.25 ± 4.68	59.22 ± 6.83	87.16 ± 2.65
Treatment	39.45 ± 8.11^*∗*^	83.73 ± 5.11^*∗*^	97.35 ± 1.29^*∗*^

^*∗*^The healing rate of the treatment group was compared with the control group at varied time points, *p* < 0.05.
